# Using reporting guidelines to publish paediatric research

**DOI:** 10.1136/archdischild-2016-311248

**Published:** 2016-10-08

**Authors:** Katie Harron

**Keywords:** Epidemiology, Evidence Based Medicine, reporting guidelines

Many medicines and devices used for the healthcare of children are unlicensed and untested for use in paediatrics, and clinicians often have to rely on evidence in adults that may not be generalisable to children.^1^ ^2^ There are a number of reasons why evidence in adults cannot always be safely extrapolated to children, including different pharmacokinetic and pharmacodynamic processes, and drug safety and efficacy being dependent on stage of development. Growing recognition of these issues has led to initiatives to increase the number of paediatric trials.^3^ In addition, recognition of the important differences in design and interpretation of trials conducted in adults and children—including ethical issues, validity of outcomes, age-specific and developmental stage-specific harms and confounders—has highlighted deficiencies in the quality of paediatric trial conduct and reporting and prompted repeated calls for child-specific reporting guidelines.^4^

Reporting guidelines such as Consolidated Standards Of Reporting Trials (CONSORT) and STrengthening the Reporting of OBservational studies in Epidemiology (STROBE) aim to improve transparency, allowing identification of potential biases, critical assessments of robustness and replication in different settings.^5^ ^6^ Many leading journals actively endorse reporting guidelines and refer authors to the Enhancing the QUAlity and Transparency Of health Research (EQUATOR) Network website (http://www.equator-network.org). The EQUATOR Network was established to improve the reliability and usability of health research literature by facilitating accurate and complete reporting of research studies.^7^

Despite the comprehensive collection of existing resources and reporting guidelines available on the EQUATOR website, there has been a lack of guidance for the reporting of paediatric studies until now.^8^ Aiming to fill this gap, and in response to the need to improve quality in reporting of paediatric research, several child-specific extensions to established guidelines are under development, including for CONSORT, Standard Protocol Items for Randomised Trials (SPIRIT) and Preferred Reporting Items for Systematic reviews and Meta-analysis (PRISMA).^5^ ^8–10^ These initiatives will complement previous guidelines by recommending consideration of paediatric-specific issues, including choice of appropriate outcomes, stratification by age or development, dosing or formulation, safety and ethical considerations. For example, detailed reporting of the age distribution of study participants is vital for understanding outcomes, treatment effects and potential effects of growth and maturation; reporting the validity of outcome measures in paediatric populations is also important, as valid outcomes for adult populations may not be relevant across childhood.^11^ Reporting long-term safety outcomes is also required in situations where harms may appear later on in development.

The most informative reporting guidelines are underpinned by robust methodological development, typically through establishing consensus from experts and stakeholders in an iterative process of feedback and review. However, providing robust evidence about the impact of guidelines on quality of reporting is challenging.^12^ ^13^ The list of 320 reporting guidelines currently published on the EQUATOR website is continuing to grow (as of July 2016), and while some argue that these checklists represent another hurdle to publication, others recognise that any tool to improve the quality and transparency of research reports can only increase the likelihood of manuscript acceptance.^14^ In addition to supporting authors in producing accurate and transparent representations of their research, reporting checklists are also a valuable aid for peer reviewers assessing the quality of studies submitted for publication.^15^ Despite the well-recognised shortcomings of peer review, including inevitable inconsistencies, limited capacity to identify all errors or weaknesses and potential reviewer biases, the current system is a crucial component of scientific research publication. Encouraging the use of reporting checklists can help to improve the process and support reviewers in providing quality reviews.^16^

Journals implement reporting guidelines in various ways, but commonly refer to relevant reporting guidelines in their Instructions to Authors. Some journals have explicit philosophies of transparency, accuracy and completeness in reporting. For example, the *BMJ* journal group's ‘Transparency Policy’ requests that authors follow complete reporting checklists prior to submission (http://www.bmj.com/about-bmj/resources-authors/forms-policies-and-checklists/transparency-policy). For *Archives of Disease in Childhood*, as for other *BMJ* journals, authors are referred to the EQUATOR Network website and encouraged to use appropriate guidelines to “ensure that you provide enough information for editors, peer reviewers and readers to understand how the research was performed and to judge whether the findings are likely to be reliable”. Other journals, such as *PLoS Medicine*, require authors to submit appropriate checklists alongside their manuscript or to provide an explanation if no relevant guideline exists.

Given the challenges in conducting randomised trials in children, observational studies based on population-based administrative data sources are increasingly being used to provide evidence and support quality improvement for paediatrics. Administrative or electronic health data sources contain individual-level records primarily collected for reasons other than research (eg, financial or clinical management) and can provide rich, detailed information on patient pathways.^17^ However, there are unique challenges for the analysis of such data.^18^ Administrative data do not always contain the complete, accurate information that researchers require. For example, a study of children with and without diabetes requires accurate classification of the disease, which is reliant on (i) the clinician recognising the diagnosis, (ii) the diagnosis being recorded in clinical notes, (iii) medical coders correctly coding the diagnosis and (iv) researchers including the correct codes in their analysis. Omissions in any of these steps could lead to missing information, which could in turn lead to bias. Transparency of reporting is therefore key to producing valid and reliable research based on administrative data. The REporting of studies Conducted using Observational Routinely-collected health Data (RECORD) initiative aims to complement the STROBE guidelines by providing guidance on issues relating specifically to administrative data, including the use of data linkage, access and availability of data and code list validation.^19^ For example, RECORD recommends that algorithms or codes used to identify the study population, exposures, outcomes and other variables are listed in detail; that any filtering based on data quality, data availability or linkage should be described and that the implications of using data not collected specifically for research should be discussed.^19^

Positive public perception of paediatric research is crucial, both in facilitating recruitment of children into trials and in exploiting existing data sources for child health research. Both authors and journals have an important role to play in supporting the public in making informed decisions about the use of their data, through maintaining a high level of transparency and quality in reporting of paediatric health research. The use of reporting guidelines by authors and reviewers can aid careful interpretation of findings and enable those working in health policy to make evidence-based decisions on paediatric care and health systems.

## References

[1] Magalhães J, Rodrigues AT, Roque F, et al Use of off-label and unlicenced drugs in hospitalised paediatric patients: a systematic review. Eur J Clin Pharmacol 2015;71:1–13. 10.1007/s00228-014-1768-9 25318905

[2] Klassen TP, Hartling L, Craig JC, et al Children are not just small adults: the urgent need for high-quality trial evidence in children. PLoS Med 2008;5:e172 10.1371/journal.pmed.0050172 18700813 PMC2504487

[3] Frakking F, van der Lee J, Klassen T, et al The StaR Child Health Project: Standard for Research with Children. Survey of current guidance for child health clinical trials 2011 http://www.who.int/childmedicines/publications/GUIDANCECHILDHEALTH.pdf (accessed 26 Jul 2016).

[4] Clyburne-Sherin AV, Thurairajah P, Kapadia MZ, et al Recommendations and evidence for reporting items in pediatric clinical trial protocols and reports: two systematic reviews. Trials 2015;16:417 10.1186/s13063-015-0954-0 26385379 PMC4574457

[5] Schulz KF, Altman DG, Moher D CONSORT 2010 statement: updated guidelines for reporting parallel group randomised trials. BMJ 2010;340:c332 10.1136/bmj.c332 20332509 PMC2844940

[6] von Elm E, Altman DG, Egger M, et al Strengthening the reporting of observational studies in epidemiology (STROBE) statement: guidelines for reporting observational studies. BMJ 2007;335:806–8. 10.1136/bmj.39335.541782.AD 17947786 PMC2034723

[7] Simera I Chapter 5.6: Reporting guidelines: a tool to increase completeness, transparency, and value of health research published in your journal. In: Smart P, Maisonneuve H, Polderman A, eds. Science Editors’ Handbook European Association of Science Editors. 2013 http://www.ease.org.uk

[8] Hartling L, Wittmeier KDM, Caldwell P, et al, StaR Child Health Group. StaR Child Health: developing evidence-based guidance for the design, conduct, and reporting of pediatric trials. Pediatrics 2012;129(Suppl 3):S112–17. 10.1542/peds.2012-0055C 22661756

[9] Liberati A, Altman DG, Tetzlaff J, et al The PRISMA statement for reporting systematic reviews and meta-analyses of studies that evaluate healthcare interventions: explanation and elaboration. BMJ 2009;339:b2700 10.1136/bmj.b2700 19622552 PMC2714672

[10] Chan AW, Tetzlaff JM, Gøtzsche PC, et al SPIRIT 2013 explanation and elaboration: guidance for protocols of clinical trials. BMJ 2013;346:e7586 10.1136/bmj.e7586 23303884 PMC3541470

[11] Sinha I, Jones L, Smyth RL, et al A systematic review of studies that aim to determine which outcomes to measure in clinical trials in children. PLoS Med 2008;5:e96 10.1371/journal.pmed.0050096 18447577 PMC2346505

[12] Turner L, Shamseer L, Altman DG, et al Does the CONSORT Statement influence the quality of reporting of RCTs published in medical journals? A Cochrane review. *Syst Rev* 2012;1:60. doi: 10.1186/2046-4053-1-60, MR000030 10.1002/14651858.MR000030.pub2 PMC3564748 23194585

[13] Moher D, Jones A, Lepage L Use of the CONSORT statement and quality of reports of randomized trials: a comparative before-and-after evaluation. JAMA 2001;285:1992–5. 10.1001/jama.285.15.1992 11308436

[14] The PLOS Medicine Editors. From checklists to tools: lowering the barrier to better research reporting. PLoS Med 2015;12:e1001910 10.1371/journal.pmed.1001910 26600090 PMC4658199

[15] Hirst A, Altman DG Are peer reviewers encouraged to use reporting guidelines? A survey of 116 health research journals. PLoS ONE 2012;7:e35621 10.1371/journal.pone.0035621 22558178 PMC3338712

[16] Cobo E, Cortés J, Ribera JM, et al Effect of using reporting guidelines during peer review on quality of final manuscripts submitted to a biomedical journal: masked randomised trial. BMJ 2011;343:d6783 10.1136/bmj.d6783 22108262 PMC3222149

[17] Jutte DP, Roos LL, Brownell MD Administrative record linkage as a tool for public health research. Annu Rev Public Health 2011;32:91–108. 10.1146/annurev-publhealth-031210-100700 21219160

[18] Hashimoto RE, Brodt ED, Skelly AC, et al Administrative database studies: goldmine or goose chase? Evid Based Spine Care J 2014;5:74–6. 10.1055/s-0034-1390027 25278880 PMC4174180

[19] Benchimol EI, Smeeth L, Guttmann A, et al The REporting of studies Conducted using Observational Routinely-collected health Data (RECORD) Statement. PLoS Med 2015;12:e1001885 10.1371/journal.pmed.1001885 26440803 PMC4595218

